# fromage: A library for the study of molecular crystal excited states at the aggregate scale

**DOI:** 10.1002/jcc.26144

**Published:** 2020-01-07

**Authors:** Miguel Rivera, Michael Dommett, Amir Sidat, Warda Rahim, Rachel Crespo‐Otero

**Affiliations:** ^1^ Department of Chemistry, School of Biological and Chemical Sciences Queen Mary University of London London UK

**Keywords:** exciton, molecular aggregate, ONIOM, photochemistry, Python library

## Abstract

The study of photoexcitations in molecular aggregates faces the twofold problem of the increased computational cost associated with excited states and the complexity of the interactions among the constituent monomers. A mechanistic investigation of these processes requires the analysis of the intermolecular interactions, the effect of the environment, and 3D arrangements or crystal packing on the excited states. A considerable number of techniques have been tailored to navigate these obstacles; however, they are usually restricted to in‐house codes and thus require a disproportionate effort to adopt by researchers approaching the field. Herein, we present the FRamewOrk for Molecular AGgregate Excitations (fromage), which implements a collection of such techniques in a Python library complemented with ready‐to‐use scripts. The program structure is presented and the principal features available to the user are described: geometrical analysis, exciton characterization, and a variety of ONIOM schemes. Each is illustrated by examples of diverse organic molecules in condensed phase settings. The program is available at https://github.com/Crespo-Otero-group/fromage.

## Graphical abstract

Accurately modeling the photoexcitations of molecular aggregates involves complementing excited state methodologies with auxiliary calculations. Due to the diversity of chemical and structural space in multimolecular complexes, these calculations require a high degree of adaptability which calls for the use of modular programming tools hitherto unavailable for the investigation of photochemical properties of molecular aggregates. The FRamewOrk for Molecular AGgregate Excitations (fromage), described herein, compiles geometry manipulation tools, exciton state characterization, and QM:QM′ models for geometry optimization.

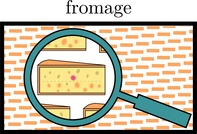

## 1 INTRODUCTION

The computational study of photochemistry in molecular condensed phases represents a notoriously difficult problem. The increased computational cost associated with accurate excited state methods is compounded by the need for larger system sizes in these materials.

In the case of molecular crystals, bound by van der Waals (vdW) forces, photochemical phenomena are thought not to delocalize beyond the immediate vicinity of the absorbing molecule.^1^ This renders full‐unit cell's periodic excited‐state property calculations less pertinent than they would initially seem. In fact, the structural features, which are relevant to these systems, are of the scale of the aggregate, ranging from monomer conformation and dimer packing all the way to molecular cluster shape.

There is therefore a potential overlap in methodology between finite size molecular aggregates and periodic molecular crystals when dealing with excited state processes. The traditional approach to investigating molecular crystals—using periodic electronic structure codes—should thus be complemented by adequately extending molecular aggregate methods for the excited state.

The generation and analysis of these aggregate nuclear configurations is often separated from the codes that produce their corresponding electronic structure. While the former tasks are typically less computationally demanding than the latter one and therefore might be added as an auxiliary feature to a larger code (e.g., Crystal17^2^), they often require a degree of flexibility which situates them more comfortably in the realm of the programming library. Indeed the bridging of scales between the periodic crystal, finite cluster, dimer, and monomer are challenging to generalize due to the formidable conformational variety of organic molecules.

An optimal strategy is therefore to provide modular tools for the investigator to tailor to their system, accompanied by ready‐made scripts that compile those tools for use in ubiquitous cases. In this way, nonexpert users are able to use the program's principal features with a reliable degree of robustness, while more comfortable users can repurpose and extend the code to better suit fringe cases.

**Scheme 1:**
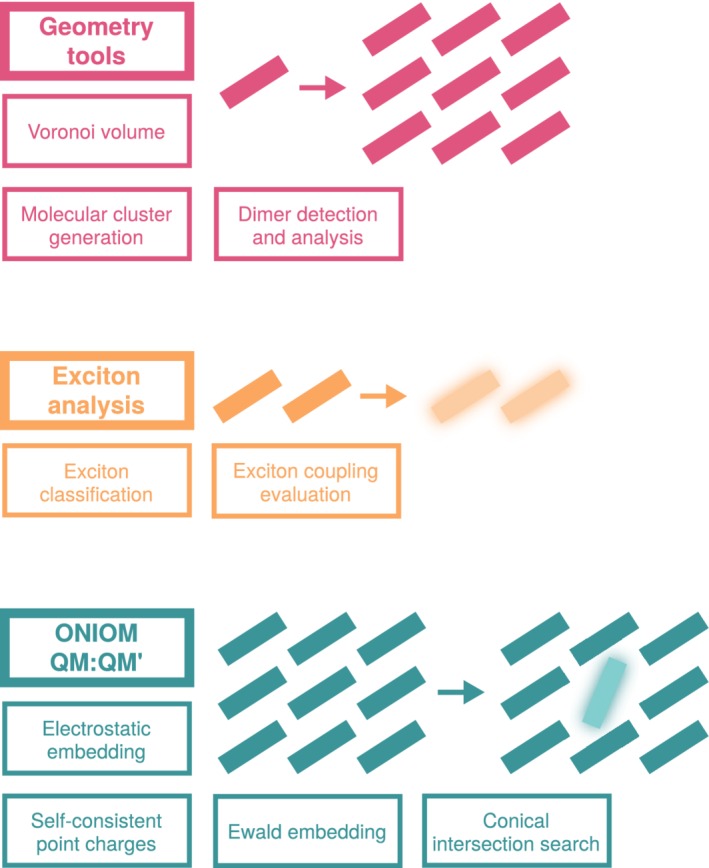
Principal features of fromage [Color figure can be viewed at http://wileyonlinelibrary.com]

There exists a variety of computational chemistry scripting libraries for different tasks, with the ecosystem in rapid development. Their promise is to optimize and standardize the research workflow for increasingly specialized operations thanks to robust, tailored tools. The Cambridge Structural Database Python API^3^ focuses on crystallographic property analysis, accessing its associated database. The Atomic Simulation Environment (ASE)^4^ specializes in interfacing with numerous electronic structure codes and communicates with them via Python scripting or a graphical user interface. RDKit^5^ provides programming tools for general‐purpose chemoinformatics and is itself used in numerous child libraries. Chemshell provides additive QM:MM interfaces between electronic structure and force field codes.^6^ Libra is a library designed for the development of quantum and classical dynamics.^7^ To our knowledge, there still lacks a library dedicated to the examination of photochemistry in molecular aggregates and crystals, exploiting the overlap in methodology between the two materials. To address this, we offer the FRamewOrk for Molecular AGgregate Excitations (fromage). fromage is a standalone Python library, accompanied by ready‐to‐use command line scripts destined to facilitate the study of molecular aggregates in the excited state. They are summarized in Scheme 1. The program is tested for Python 2.7 and 3.6, though the authors do not guarantee backwards compatibility with Python 2 in future releases. For geometry manipulation routines, the program only relies on the unit cell's Cartesian structure and lattice vectors. From this information, the user can obtain unique dimer configurations, molecular clusters and general structural information.

All of the electronic structure calculations are performed by popular quantum chemistry programs. Currently, interfaces are provided to run calculations in DFTB+,^8^ Gaussian,^9^ Molcas,^10^ and Turbomole.^11^ By delegating these calculations to different programs, their results can be combined into hybrid energy expressions. Thus fromage can perform Our own N‐layered Integrated molecular Orbital and Molecular mechanics (ONIOM) calculation while taking advantage of the diversity of modeling methods of several programs instead of only one. The electrostatic embedding in these calculated can be extended to include the Coulomb interaction of the whole crystal by fitting point charges to an Ewald potential.^12^

fromage has been used to study various Quantum Mechanics in Quantum Mechanics (QM:QM′) electrostatic embedding schemes for applications in photoactive molecular crystals,^12^ the aggregation‐induced emission process in propeller‐shaped molecules,^13^ and the design principles for proton transfer luminescent materials.^14^

In this article, we present an overview of fromage and its capabilities. First, we describe the program structure, illustrated by basic usage examples. Then, we enumerate its principal features, namely, geometric analysis, exciton coupling evaluation, and ONIOM QM:QM′ models.

## 2 PROGRAM STRUCTURE

### 2.1 Principal classes

fromage makes use of two main classes in most of its operations, Atom and Mol. An Atom object is defined as a point in Cartesian space with associated physical properties. If the point is to represent an atom, supplying its element string provides standard properties such as covalent radius or atomic mass. The partial charge can also be specified, which can become useful in representing both atoms and point charges.

**Figure 1:**
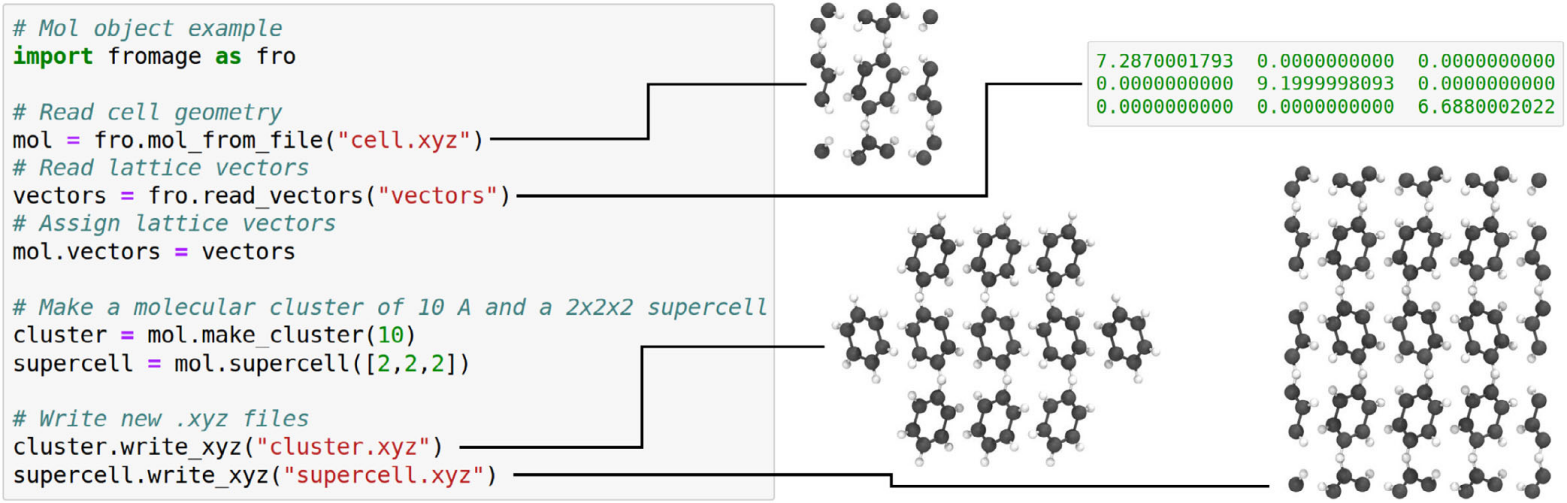
Example of use of fromage as a Python library. The three dimensional structures are shown in orthographic projection [Color figure can be viewed at http://wileyonlinelibrary.com]

In practice, the user has little direct interaction with Atom objects. Instead, they manipulate Mol objects, which contain a list of the former. Mol extends common Python list methods via composition, allowing for intuitive appending, indexing, iterating, and so on, but also provides methods to manipulate molecular aggregate geometries and unit cells. Mol objects can be created explicitly or generated from a typical geometry file such as .xyz. For instance, one may generate a molecular cluster and a supercell from unit cell information as illustrated in Figure 1.

Apart from the constituent atoms and lattice vectors, Mol also has attributes pertaining to the definition of a bond within the collection of atoms. Two atoms are said to be bonded when their distance falls below a certain threshold mol_thresh. This distance can be measured from nucleus to nucleus but also from the edge of the spheres of vdW radius or covalent radius. The method is selected by varying the mol_bonding attribute. Having this flexibility is required for highly distorted molecular geometries or diverse element combinations. Armed with the definition of a bond, the Mol class can single out covalently bonded complexes from an aggregate, generate molecular clusters from a single crystal, and detect atomic connectivity.

These tools are in and of themselves useful as a library for the Python literate user. However, several ready‐made scripts are supplied for more complicated procedures and frequently required operations. Of the most practical use is perhaps fro_uc_tools.py, a command line script, which performs operations related to unit cells. It is essential for comfortably communicating between periodic and finite systems which is a central concept in fromage. For instance, given a unit cell geometry file cell.xyz and a text file with the lattice vectors, the line:

fro_uc_tools.pycell.xyzvectors−r15

will produce a file cluster_out.xyz containing the geometry of a cluster of whole molecules where all atoms lie within a radius of 15 Å from the origin.

**Figure 2:**
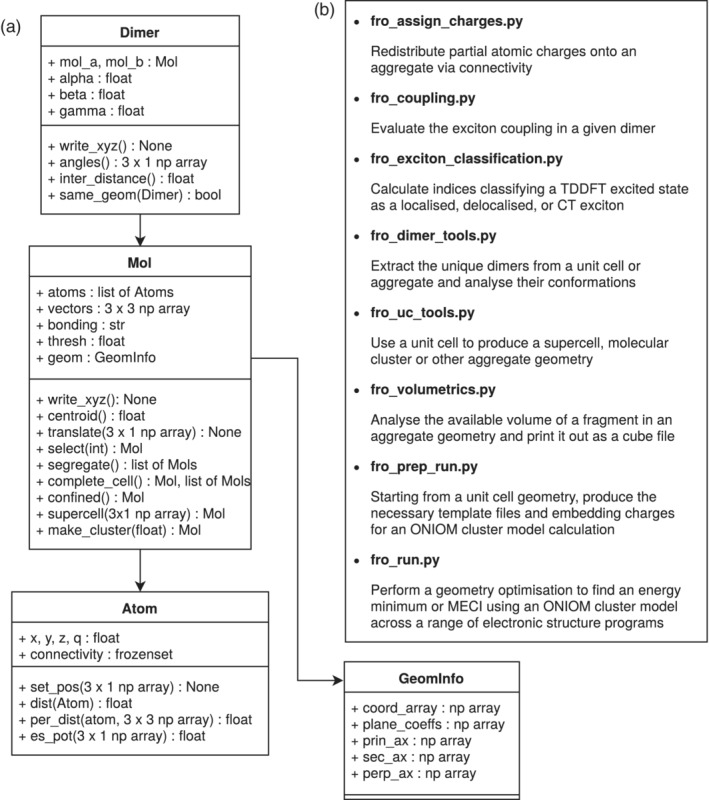
(a) Principal class diagram of fromage. (b) Description of the main callable modules

The other scripts are used to operate on unit cells, clusters, dimers, and monomers. They are discussed in Section 3 and represented in Figure 2 along with the core class structure.

### 2.2 Dependencies

The most common calculation performed by the geometry manipulation routines is the evaluation of interatomic distances. Therefore, for an overall speedup, these distances are calculated in C++ and wrapped as a Python function using the Simplified Wrapper and Interface Generator (SWIG). Some other more involved operations are sped up by using the numpy^15^ library. Geometry optimization is carried out with the Broyden–Fletcher–Goldfarb–Shanno (BFGS) implementation in scipy.^16^

The program Ewald by Derenzo et al.^17^, ^18^ is used for the fitting of point charges to the Ewald potential. It is modified to allow the use of partial charges and redistribution with permission from the authors here https://github.com/Crespo-Otero-group/Ewald.

## 3 FEATURES

### 3.1 Geometrical analysis

#### 3.1.1 *Voronoi volumes*

The conformational freedom of a molecule in the gas phase is only limited by its structural features and how they relate to the potential energy surfaces (PESs) of particular reaction coordinates. The electronic excitations within a molecule can in general be rationalized by scrutinizing and comparing its different PESs. In contrast, in condensed phases, a molecule's freedom of nuclear reorganization is hindered by the close packing imposed by its environment. The study of the effect of this close packing on excited‐state PESs and photochemical behavior is a vast topic and involves an accumulation of inter‐related factors both electronic and nuclear in nature which are often hidden behind the deceptively concise term of *steric hindrance*.^19^, ^20^

It is, however, appropriate to begin such an investigation by obtaining computationally inexpensive and easily interpretable features of the packing of the aggregate. A routine approach for crystals is dividing the unit cell volume by the amount of molecules in the cell to find the average volume *V*_c_ assigned to the molecule within the packing pattern in order estimate the tightness of packing. However, this volume is difficult to compare between crystals composed of different molecules and is not visualizable. The determination of Voronoi volumes for molecules in aggregate is a promising alternative.

In an aggregate of atoms a point belongs to the Voronoi volume of a given molecule if the atom it is nearest to belongs to that molecule. The application of Voroni cells^21^ to molecular systems has successfully been used to characterize the geometry of condensed phases^22^ and notably liquids.^23^ This definition is refined by scaling the distance metric by the vdW radius of the atom; thus, for instance, assigning more space to oxygen than to hydrogen. For the calculation of distances, we employ a grid‐based scheme, which makes it robust and allows us to choose an arbitrary resolution for the volume.^24^, ^25^ The resulting Voronoi volume *V*_V_ can be compared to the sum of the vdW spheres of the atoms in the molecule (counting their intersection only once) *V*_vdW_ to obtain a volumetric index $V_{i}=\frac{V_{V}}{V_{\mathrm{vdW}}}$, which gives a normalized indication of the tightness of packing in the crystal for a specific molecule.

The Voronoi volumes introduce a distinction between inequivalent molecules in a crystal, and in fact should average out to *V*_c_ if the *V*_V_ values are weighted by the amount of occurrences of the molecule per unit cell. They can be used in finite systems such as amorphous clusters and perhaps most importantly, they are visualizable. Seeing the shape of a Voronoi volume can indicate the available space in the aggregate and therefore the areas of the PES least restricted by the environment.

A user may open a cluster geometry file clust.xyz with a visualizing program and choosing which molecule they wish to calculate the Voronoi volume of with fromage. They identify the molecule by marking down the label of any atom belonging to the molecule. Then, upon calling:

$$\mathrm{fro}\_\text{volumetrics}.\mathrm{py}\;\text{clust}.\mathrm{xyz}-l\;\left[\text{atom label}\right]$$

the program will generate the files voro.cube, vdw.cube, and union.cube, which are the visualizable Voronoi and vdW volumes of the molecule, and their union (in the set theory sense). A file called volumes contains the integrated volume of each of these.

**Figure 3:**
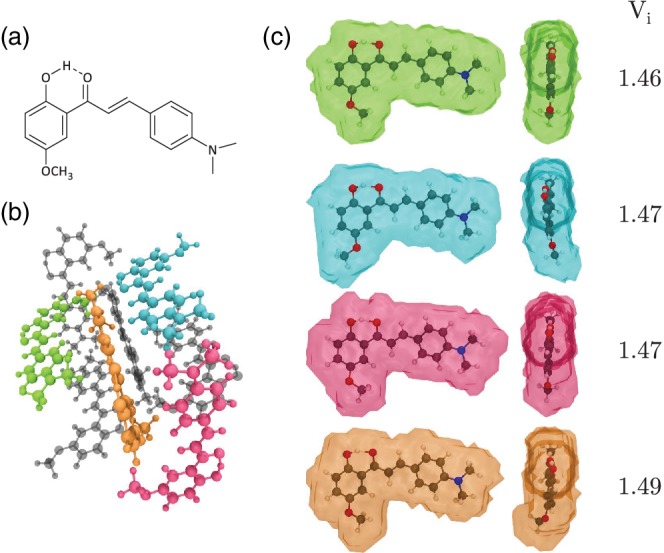
(a) Chemical structure of DAP, (b) unit cell of the DAP crystal with the asymmetric cell highlighted by color, (c) profiles of the Voronoi volumes for the four molecules of the asymmetric cell with their volume index. DAP, (E)‐1‐(2‐hydroxy‐5‐methoxyphenyl)‐3‐(4[dimethylamino]phenyl)prop‐2‐en‐1‐one [Color figure can be viewed at http://wileyonlinelibrary.com]

An illustrative example is a derivative of 2′‐hydroxychalcone (HC), which upon the addition of a methoxy group in *para* position with respect to the hydroxyl group turns off its emissive character in crystal form, bringing the fluorescence quantum yield from 0.32 to less than 0.01.^26^, ^27^ The new molecule is (E)‐1‐(2‐hydroxy‐5‐methoxyphenyl)‐3‐(4[dimethylamino]phenyl)prop‐2‐en‐1‐one (DAP). While HC exhibits herringbone style packing, DAP has a complex unit cell structure whose steric constraints on the individual molecules are unclear at first glance. Figure 3 shows the difference in tightness of crystal packing between inequivalent monomers of DAP.

#### 3.1.2 *Dimeric arrangement*

Excitations in molecular aggregates are not guaranteed to remain confined to one absorbing monomer. Indeed, the electronic wavefunctions of the neighboring molecules may have enough overlap to produce intermolecular electronic interferences in the excited state. This can manifest in all sorts of photochemical processes and is central to exciton‐governed mechanisms like charge transfer or singlet fission. In crystals, typical packing motifs like herringbone or sheet‐like, produce a limited set of archetypal dimer arrangements such as edge‐to‐face or face‐to‐face which have generalizable excitonic behavior for chemically similar molecules.^14^, ^28^ For instance, in face‐to‐face aromatic systems, π–π interactions are a defining feature of the excitonic states.

Regardless of whether a researcher is investigating an amorphous cluster or a crystal structure, it is therefore informative to extract all of the significant dimers in the system and to quantify their geometrical arrangement in order to classify them under the principal dimer archetypes at a glance. In fromage, the user can extract the possible dimers whose distance falls below a given threshold. The intermolecular distance can be defined either as the centroid‐to‐centroid distance or as the nearest intermolecular atom pair distance. The latter can also be complemented by the vdW radii of the atoms. If the molecules are in lattice positions, the symmetry of the unit cell will produce groups of dimers identical up to a reflection or rotation. To filter out repeated configurations, all the intermolecular atomic distances are evaluated and sorted which provides a fingerprint for the dimer geometry. Equivalent dimers are then defined as ones with the same fingerprint up to a root‐mean‐squared deviation threshold of 10^−4^ Å by default.

The dimeric arrangement can then be characterized quantitatively. An orthonormal set of principal, secondary, and tertiary axes is calculated for each constituent fragment, and the angles between same axes of two molecules can be associated to an archetypical dimer, effectively classifying the pair.

**Figure 4:**
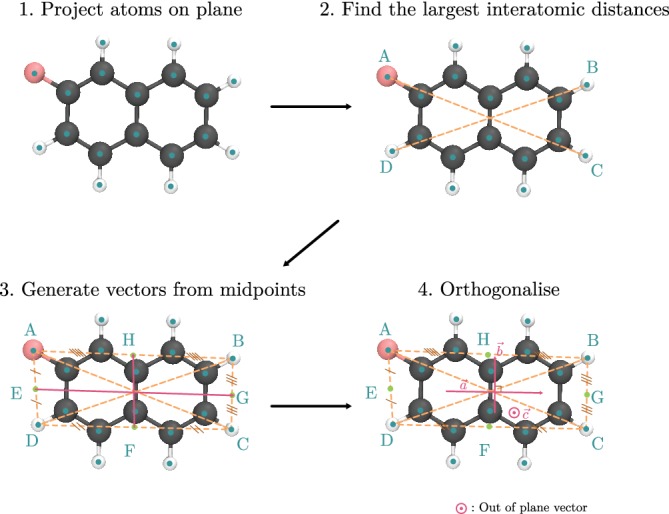
Algorithm to generate principal axes from a monomer geometry with 2‐fluoronaphthalene as an example [Color figure can be viewed at http://wileyonlinelibrary.com]

The generalized procedure to obtain characteristic vectors for a molecule is shown in Figure 4:

1. All atoms of the molecule are projected onto an averaged plane by singular value decomposition.
2. The two longest interatomic distances are identified, forming a quadrilateral *ABCD* such that *AC* > *BD* and *AB* is the longest side. This imposes an arbitrary but consistent direction for the vectors.
3. The following midpoints are detected: [*AB*] → *H*; [*BC*] → *G*; [*CD*] → *F*; [*DA*] → *E*. The principal and secondary vectors $\vec{a}$ and $\vec{b}$, respectively, go from *E* to *G* and *F* to *H*.
4. The two vectors are normalized and rotated equally until they are perpendicular. The tertiary vector is $\vec{c}=\vec{a}\times \vec{b}$.

This orthonormal set of axes presumes the significance of a plane which defines the shape of the molecule. Conjugated organic systems often have most atoms in one plane, and in other cases, the judicious elimination of extrenuous atoms from the analysis—a feature present in the code—can reduce the significant coordinates to a plane. To exclude a particular atom type from the calculation, only one atom need be specified, and others with the same identity can be automatically detected using the method outlined in Section 3.3.4.

If one same atom is involved in both largest interatomic distances, the procedure remains valid and the Points *B* and *C* become degenerate. In certain cases, the principal axis should simply be the vector connecting the largest interatomic distance. When this option is selected, the secondary axis becomes the perpendicular axis which lies on the averaged plane. In the general case where one wishes to extract the geometric information from a cluster of dimers clust.xyz, one should use the command:

fro_dimer_tools.pyclust.xyz,

which will return the file dimers.dat containing all of the angles between dimers, the centroid distances and a proposed classification into different archetypical dimers. An additional slip angle is printed, to estimate the amount of face‐to‐face overlapping area giving rise to π–π interactions. It is defined as the smallest angle between the centroid‐to‐centroid axis and either tertiary axis of the constituent monomers, as is discussed by Dommett et al. 14.

**Figure 5:**
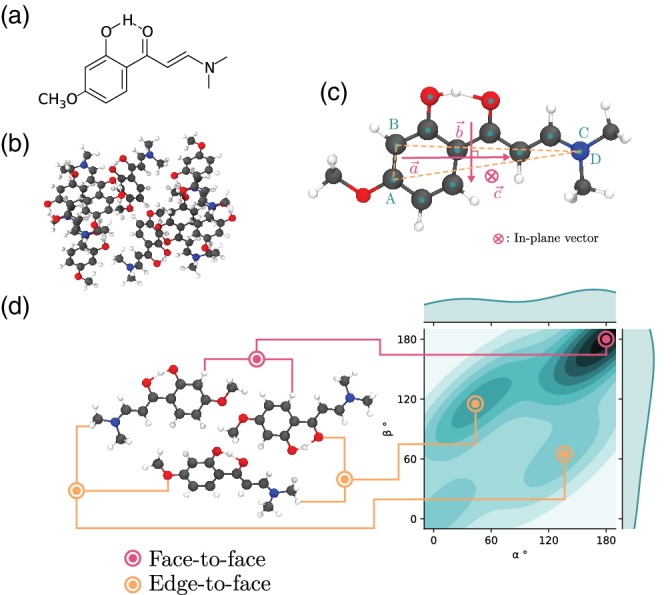
Case study of DMAH for dimer geometry analysis: (a) chemical structure, (b) crystalline cluster, (c) principal axes determination, and (d) heat map of the angles between primary and secondary axes, respectively, *α* and *β*. DMAH, (2E)‐3‐(dimethylamino)‐1‐(2‐hydroxy‐4methoxyphenyl)‐2‐propen‐1‐one [Color figure can be viewed at http://wileyonlinelibrary.com]

The above method was used to investigate (2E)‐3‐(dimethylamino)‐1‐(2‐hydroxy‐4methoxyphenyl)‐2‐propen‐1‐one (DMAH). This molecule exhibits lasing behavior with a fluorescence quantum yield of 0.77 in crystal form compared to 0.19 in polymethylmethacrylate film.^29^ The crystal structure was optimized in Quantum Espresso using PBE‐D2 with a plane‐wave cutoff of 30 Ry and a 8 × 6 × 6 *k*‐point mesh. A cluster of molecules was extracted from its crystal positions and all dimers with centroids falling less than 10 Å from each other were considered. In this case, certain nonessential atoms were ignored in the geometric analysis and the Points *B* and *C* became degenerate. The results are represented on Figure 5. The points at (44°, 115°) and (136°, 65°) have a large *β* value, which is characteristic of the edge‐to‐face dimers in herringbone packing. However, the *α* values are unusually far from 0° or 180°, showing a packing arrangement specific to this crystal. The point at (0°, 0°) is in this case only related to the dimers which monomers form with periodic images of themselves.

### 3.2 Exciton analysis

#### 3.2.1 *Exciton classification*

The exciton model is a framework to characterize different types of many‐body electronic excitations in collections of molecules. The excitation can be associated with a localized Frenkel exciton, or a charge‐transfer state, which are characterized by the electron density, respectively, migrating intramolecularly and intermolecularly. A delocalized Frenkel excitation corresponds to electron density reorganization throughout the dimer with no net charge transfer. Differentiating between these three behaviors is crucial for the design of organic semiconductors in solar cells, because the process of charge separation and migration is fundamental to their mechanism. One approach is that of analyzing the one‐electron transition density matrices associated with the excitation, which contains information about the migration of the charge during the process.^30^ This method is implemented in TheoDORE.^31^

Crespo‐Otero and M. Barbatti^32^ and Sen et al.^33^ propose a different scheme to qualify the nature of the exciton by comparing the charge density distribution on fragments of a dimer before and after excitation. The original implementation is in a program named CALCDEN, which combines Perl and Fortran. The reimplementation in fromage is Python importable, making it an attractive alternative for use and extension depending on the user's preference.

A Mulliken partition scheme can supply integrated orbital‐specific densities located on one molecule (*A*):

(1) $$\rho_{A}^{k}=\sum_{\mu \in A,\nu \in A}c_{\mathrm{\mu k}}c_{\mathrm{\nu k}}S_{\mu \nu }+\sum_{\mu \in A,\nu \in B}c_{\mathrm{\mu k}}c_{\mathrm{\nu k}}S_{\mu \nu },$$

where *S*_*μν*_ is the overlap integral between basis functions *ϕ*_*μ*_ and *ϕ*_*ν*_, and *c*_*μk*_ is the coefficient of basis function *ϕ*_*μ*_ in molecular orbital *k*. This density, $\rho_{A}^{k}$, can be used to produce two indices related to an excitation *I*:

(2) $$\sum P_{A}^{I}=\sum_{i\to j}\sigma_{\mathrm{ij}}{\left(C_{i\to j}^{I}\right)}^{2}\left(\rho_{A}^{j}+\rho_{A}^{i}\right),$$

$$\Delta P_{A}^{I}=\sum_{i\to j}\sigma_{\mathrm{ij}}{\left(C_{i\to j}^{I}\right)}^{2}\left(\rho_{A}^{j}-\rho_{A}^{i}\right),$$

where $CC_{i\to j}^{I}$ is the Time‐Dependent Density Functional Theory (TDDFT) coefficient corresponding to the excitation from orbital *i* to *j* and *σ*_*ij*_ is 1 for *i* < *j* and −1 for *i* > *j*. These indices are in units of e^−^ and since they are associated with one excited state only, they have bounds *0* ≤ $\sum P_{A}^{I}$ ≤ 2 and −1 ≤ $\Delta P_{A}^{I}$ ≤ 1.

The combination of the two quantities indicates the behavior of the electronic density upon excitation; a large reorganization of density confined to Molecule *A* (labeled LOC(A)) would manifest in an extreme $\sum P_{A}^{I}$, closer to 0 e^−^ for Molecule *B* and closer to 2 e^−^ for Molecule *A*. On the other hand, extreme $\Delta P_{A}^{I}$ values indicate a net loss or gain of density by one molecule. Values closer to −1 e^−^ correspond to a charge transfer from *A* to *B* (CT(A→B)) and vice versa for values close to 1 e^−^. If none of the indices have extreme values, the excitation is delocalized, labeled DELOC. In fromage, an arbitrary threshold is in place by default where an excitation less than 0.5 e^−^ from an extreme value is classified as the corresponding type of exciton.

To evaluate the two indices and classify an excitation, the user should first perform a TDDFT or Configuration Interaction Singles (CIS) calculation of the dimer. Currently, only Gaussian calculations are supported. They should ensure that the atoms of molecule *A* appear before those of *B* in the geometry field and that an rwf file is produced by using the option %rwf = [name].rwf. Then, given the output files tddft.log and tddft.rwf, the command line:

$$\mathrm{fro}\_\text{exciton}\_\text{classification}.\mathrm{py}\;\text{tddft}.\log\;\text{tddft}.\mathrm{rwf}\;\left[\text{number of excitation}\right]$$

will print out the values of $\sum P_{A}^{I}$ and $\Delta P_{A}^{I}$ along with a suggested classification (DELOC, LOC(A), LOC(B), CT(B→A), and CT(A → B)).

**Figure 6:**
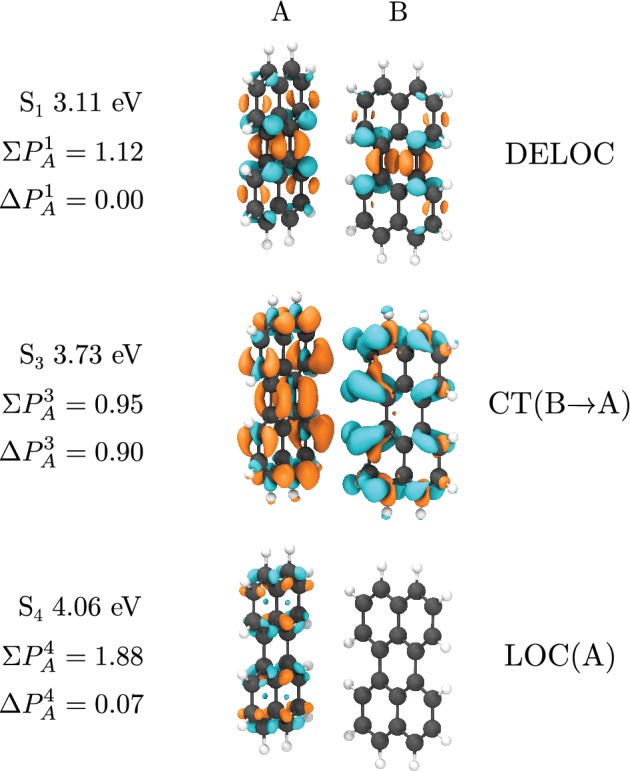
Transition density of two excited states of a perylene dimer taken from its crystal structure. The excited state energies are reported with respected to *S*_0_. The electron density migrates from the blue to the yellow areas upon excitation. The exciton classification indices are defined in Equation (2) and are in units of e^−^ [Color figure can be viewed at http://wileyonlinelibrary.com]

To illustrate the use of this feature, a dimer of perylene was extracted from its experimental crystal structure.^34^ Its excited states were calculated in Gaussian16 using TD‐ωB97X‐D/ 6‐31G(d), and the transition densities analyzed using the orbital specific Mulliken partition scheme. The states *S*_3_ and *S*_4_ each had extreme values of the classification indices, with $\sum P_{A}^{3}$ = 0.95 e^−^ and $\Delta P_{A}^{3}$ = 0.90 e^−^ for the former, indicating a charge transfer from Fragment B to A, and $\sum P_{A}^{4}$ = 1.88 e^−^ and $\Delta P_{A}^{4}$ = 0.07 e^−^ for the latter, corresponding to a transition confined to Monomer A. Figure 6 corroborates this classification by showing the electronic transition density of a DELOC S_1_ and comparing it to the CT(B → A) S_3_ and LOC(A)S_4_.

#### 3.2.2 *Exciton coupling evaluation*

The coupling associated with an exciton is a measure of the correlation between the individual isolated excitations within the exciton. Evaluating it can give us a quantitative bearing on the importance of the many‐body effects in the excited state process.

Kasha's exciton model is the initial approach, reducing the electronic densities of the fragments to point dipoles and comparing their relative geometry.^28^ Under the point dipole approximation (PDA), the exciton coupling between fragments *i* and *j* is expressed as

(3) $$J_{\mathrm{ij}}=\frac{\mu_{i}\mu_{j}}{R^{3}}-\frac{3\left(\mu_{i}\cdot R_{\mathrm{ij}}\right)\left(R_{\mathrm{ij}}\cdot \mu_{j}\right)}{R^{5}},$$

where *μ*_*n*_ is the electronic transition dipole moment (TDM) for monomer *n* and *R*_*ij*_ is the vector connecting the centroids of monomers *i* and *j*.

For a better spatial resolution of the electrostatic interactions, one can instead calculate the interaction between atomic transition charges (ATC):^35^

(4) $$J_{\mathrm{ij}}=\sum_{a}^{N_{i}}\sum_{b}^{M_{j}}\frac{q_{a}q_{b}}{\left|R_{a}^{i}-R_{b}^{j}\right|},$$

where $R_{c}^{k}$ is the position of atom *c* of monomer *k* with ATC *q*_*c*_ and *N*_*k*_ is the number of atoms of monomer *k*.

For more resolution, the transition electronic density itself can be used, yielding costly but exact Coulombic exciton coupling. Additional correction can be included, for example, by including the dielectric response of the environment as a polarizable continuum model as is done in the EXcitonic Analysis Tool (EXAT).^36^

These models are purely Coulombic in nature and do not take into account the short‐range interaction affecting the excited state behavior of adjacent molecules, which, for example, is dominant in charge‐transfer states.

Aragó and Troisi have devised a procedure which evaluates the coupling *J*_*ij*_ in a dimer by diabatising the Hamiltonian:^1^

1. Select an excited state property. For the sake of argument, we will use the TDM *μ* but anything bearing relation to the excited electronic density would be applicable.
2. Evaluate the property for the two lowest excited states, along with the energies, for the dimer. These are the adiabatic energies ($E_{1}^{A}$ and $E_{2}^{A}$) and adiabatic TDMs ($\mu_{1}^{A}$ and $\mu_{2}^{A}$).
3. Evaluate the property for both isolated constituent monomers in the first excited state, retaining their orientation. These TDMs are labeled $\mu_{1}^{\mathrm{ISO}}$ and $\mu_{2}^{\mathrm{ISO}}$.
4. Calculate the singular value decomposition (***μ***^*A*^)^*^μ^ISO^ *= U*∑V^*^.
5. Compute the matrix ***C =*** (UV^*^)^*^, which is the best unitary transformation matrix mapping the adiabatic to the diabatic basis.
6. Compute the diabatic Hamiltonian:
  $$\left(\begin{array}{cc} E_{i}^{D} & J_{\mathrm{ij}}\\ J_{\mathrm{ji}} & E_{j}^{D} \end{array}\right)=\left(\begin{array}{cc} C_{11} & C_{12}\\ C_{21} & C_{22} \end{array}\right)\left(\begin{array}{cc} E_{i}^{A} & 0\\ 0 & E_{j}^{A} \end{array}\right)\left(\begin{array}{cc} C_{11} & C_{21}\\ C_{12} & C_{22} \end{array}\right).$$

The off‐diagonal elements of the Hamiltonian the exciton coupling values *J*_*ij*_. More details can be found in ref. 1. The diabatization method has already been employed in fromage to investigate the aggregate behavior of propeller‐shaped emitters.^13^

We have further extended it to calculate *N*‐dimensional diabatic Hamiltonians, thus, for example, allowing for the calculation of pairwise exciton coupling within a trimer in the excited state, taking into account the influence of the third monomer.

An additional approximate method is that of exploiting the exciton energy splitting of the molecular excited state upon formation of a dimer. In the dimer, the S_1_ and S_2_ states are separated by twice the magnitude of the exciton coupling, provided that the individual constituent molecules are in perfect resonance.^37^ Even in less symmetric cases, this approximation has been used with reasonable success.^14^, ^38^, ^39^ fromage implements the half‐gap method, the PDA Kasha model, the ATC Coulombic interaction model, and the diabatization scheme using either ATCs or TDMs as excited state properties.

The script fro_coupling.py manipulates Gaussian log files to compute exciton couplings. Several schemes are implemented. For instance, if a user requires the diabatization method using the TDM excited state property to find the diabatic Hamiltonian containing the first three couplings of a trimer, the steps would be as follows. Carry out Gaussian calculations of the S_1_ states of the three constituent monomers and the first three excited states of the trimer, yielding the files mon_1.log, mon_2.log, mon_3.log, and trim.log. Then, use the command line:

fro_coupling.py−mDIA−pTDM−mfmon_1.logmon_2.logmon_3.log−nftrim.log−ns3.

This will print out the diabatic Hamiltonian where the three lower triangular off‐diagonal elements correspond to the three exciton couplings of the trimer.

**Figure 7:**
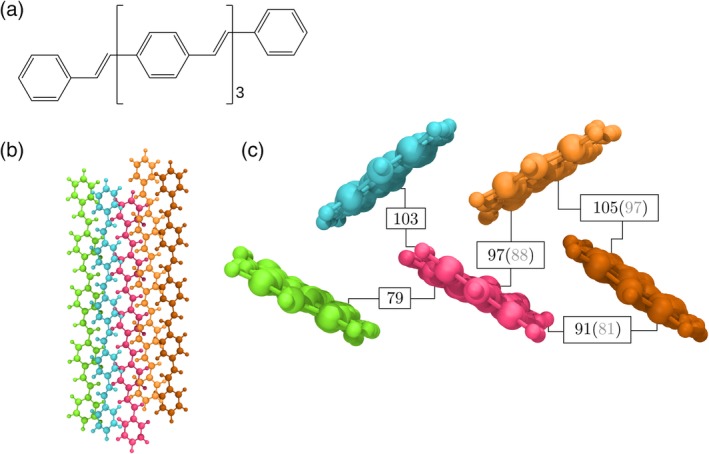
(a) Chemical structure of 4PV. (b) Top view of a cluster of 4PV molecules taken from their crystal positions. (c) Side view of the cluster with inequivalent dimers labeled by their exciton coupling value in meV. The values in parentheses are calculated as a trimer. 4PV, 1,4‐bis‐(4‐styryl‐styryl)‐benzene [Color figure can be viewed at http://wileyonlinelibrary.com]

As an illustrative example, the crystal structure of 1,4‐bis‐(4‐styryl‐styryl)‐benzene (4PV) was used to extract inequivalent dimers and evaluate their couplings. The structure of 4PV has been experimentally shown to contain six monomers, departing from the high symmetry of an ideal herringbone crystal due to variable slight rotation of the extreme phenyl rings.^40^ First, the unit cell was optimized using PBE‐D2 with a basis set cutoff of 50 Ry and a Monkhorst‐Pack grid of 1 × 2 × 1 *k*‐points as implemented in Quantum Espresso.^41^ Then, the inequivalent dimers were detected by fromage using a centroid‐centroid distance threshold of 7 Å. The TDMs of these dimers were calculated using TD‐ωB97X‐D/6‐31G(d) in Gaussian16^9^ and processed in fromage in order to evaluate exciton couplings using dimer and trimer diabatic Hamiltonians. The results are shown in Figure 7. In this system, edge‐to‐face dimer arrangements have larger exciton couplings (97, 103, and 105 meV) than face‐to‐face ones (79 and 91 meV). The largest difference is of 26 meV, which represents 25% of the greatest coupling (105 meV). It is expected that in cofacial dimers with couplings of such magnitude, the short‐range interactions should account for most of the coupling, making the use of a coupling scheme which accounts for exchange imperative.^42^

Overall, the inclusion of the third molecule in the trimer diabatic Hamiltonian reduces the magnitude of the couplings by about 10 meV which is significant since it is in the order of the difference between certain edge‐to‐face and face‐to‐face values.

The irregularities in the herringbone packing produce two different face‐to‐face dimers with respective centroid‐centroid distances of 6.05 and 6.15 Å. This relatively small increase in distance reduces the exciton coupling by 12 meV (14% of the average value), indicating that the natural packing of 4PV produces in dimers in an excitonically sensitive geometry.

### 3.3 ONIOM flavors

Calculating an excited state electronic structure is often unavoidable when evaluating an absorption or emission energy, locating nonradiative pathways, or building a PES. This often proves to be challenging due to the environmental effects in such media.

#### 3.3.1 *The ONIOM scheme*

Many approaches have been developed and tailored specifically to reflect the condensed phase nature of these materials. For periodic systems, plane‐wave basis ab initio calculations represent a tempting option due to their inherent treatment of the long‐range interactions present in molecular crystals. However, due to the often local nature of excited state phenomena in molecular aggregates, it can be instructive to treat them as defects and not to periodically repeat the excitation throughout the material.^43^, ^44^ Furthermore, their treatment from a periodic perspective implies calculating the excited‐state electronic structure of all explicit electrons in the unit cell, which, considering the relative asymmetry of molecular crystals, can represent many more electrons than are directly involved in the process.

Local ab initio theories have a longer history of methodological development for excited states, allowing for the investigation of multireference wavefunction features (CASSCF, CASPT2) and multiple excited states (ADC2, CC2). However these methods are liable to being too costly to explicitly model the environment of the excitation and must therefore rely on auxiliary corrections.

Hybrid method schemes can offer such corrections, where an active site calculated with a high accuracy method is embedded in an explicit environment of lower accuracy. Inter‐program hybrid method codes are not uncommon. GARLEEK^45^ communicates between Gaussian and Tinker for subtractive QM:MM calculations. Chemshell^6^, ^46^ provides additive QM:MM formulations. The ASE^4^ offers both additive and subtractive QM:MM as a Python library. However to our knowledge no such code yet offers inter‐program ONIOM QM:QM′ calculations in the excited state. fromage uses its interfaces with DFTB+, Gaussian, Molcas, and Turbomole to calculate energies and geometry optimizations of different kinds with ONIOM QM:QM′, namely, mechanical embedding, regular electrostatic embedding, Ewald point charge embedding, and self‐consistent versions of the last two.

ONIOM is a popular subtractive hybrid method scheme with the following energy equation:^47^, ^48^, ^49^

(5) $$E_{\text{high}:\mathrm{low}}\left(1\cup 2\right)=E_{\text{high}}\left(1\right)+E_{\mathrm{low}}\left(1\cup 2\right)-E_{\mathrm{low}}\left(1\right),$$

where Region **1** is the active site, Region **2** is its vicinity and *E*_*i*_(*n*) is the energy of region *n* calculated at the *i* level of theory. In the usual ONIOM vernacular, Region **1** is the “model system” and Regions **1** and **2** are the “real system”; however, these terms are more appropriate in the general case where the inter‐region boundary can cross bonds and link atoms are employed.^47^ For excitations in molecular aggregates, **1** contains the molecules involved in the excitation and **2** a cluster of molecules encasing them. While ONIOM was originally formulated for QM:MM hybrid calculations, the extension to QM:QM′, where QM′ is a lower accuracy method, soon followed.^48^ This variant allows for a potentially more accurate descriptions of Region **2** and the inter‐region interactions in exchange for an increased computational cost. The various quantum methods available and tested in fromage are listed in Table 1 along with their corresponding programs.

**Table 1:** Interfaced quantum methods and their availability in electronic structure programs. The acronyms are Density Functional Tight‐Binding (DFTB), Density Functional Theory (DFT), Time‐Dependent Density Fuctional Theory (TDDFT), Algebraic Diagrammatic Construction 2 (ADC2), Coupled Cluster 2 (CC2), Complete Active Space Self‐Consistent Field (CASSCF), and Complete Active Space Perturbation Theory 2 (CASPT2)

Method	DFTB+	Gaussian	Molcas	Turbomole
Hartree‐Fock	×	✓	×	✓
DFTB	✓	×	×	×
DFT	×	✓	×	✓
TDDFT	×	✓	×	✓
ADC(2)	×	×	×	✓
CC2	×	×	×	✓
CASSCF	×	✓	✓	×
CASPT2	×	×	✓	×

A further improvement to the ONIOM method stems from the use of electrostatic embedding, where the Region **1** terms of the energy equation are calculated with one‐electron point charge potential contributions in the Hamiltonian, located at Region **2** atomic sites.

For QM:QM′, the equation becomes:

(6) $$E_{\mathrm{QM}:QM^{\prime }}\left(1\cup 2\right)=E_{\mathrm{QM}}^{\mathrm{EE}}\left(1\right)+E_{QM^{\prime }}\left(1\cup 2\right)-E_{QM^{\prime }}^{\mathrm{EE}}\left(1\right),$$

where EE stands for electrostatic embedding. For QM:MM, the point charges should simply correspond to the ones used in the force field; however, for QM:QM′, the choice is up for discussion.^12^, ^50^ In the embedding of $E_{QM^{\prime }}^{\mathrm{EE}}\left(1\right)$, the type of partial charges should be motivated by the cancellation of ground state inter‐region electrostatic contributions introduced in the second term of Equation 6. Using that same set of charges for $E_{\mathrm{QM}}^{\mathrm{EE}}\left(1\right)$ has been argued to compensate overpolarization effects in ground state ONIOM calculations^51^; however, this invites a low‐resolution description of the environmental electrostatic interactions due to the stringent constraints on the computational cost of the QM′ level of theory. In fact Restricted ElectroStatic Potential (RESP) charges originating from a high level of theory population analysis should by definition provide a point charge potential closest to that of the molecular electron density. fromage allows the user to choose between RESP and Mulliken charges for all of its electrostatic embedding formulations.

#### 3.3.2 *Ewald embedding*

Whatever the choice, a serious problem arises with the use of cluster models to represent the condensed phase. Indeed environmental effects on the excitation stemming from molecules beyond the immediate vicinity of Region **1** have been completely omitted. This is acceptable for short‐range contributions to the interaction energy since they decay very quickly with distance and are therefore dominated by nearest neighbor contributions. However, the Coulomb interaction decays as an inverse law and therefore has significant long‐range contributions to the excitation, compounded by the long‐range order symmetry in the case of molecular crystals.

Simply increasing the size of Region **2** is not an adequate solution as the Madelung sum in crystals is known to be slowly and conditionally convergent, thus requiring an impractically large cluster with difficult to prove accuracy. The Ewald sum is an equation designed to closely approximate the Madelung potential with fast convergence by splitting the electrostatic potential terms into real and reciprocal space via the use of judicious Fourier transforms. The resulting potential is as follows:^52^, ^53^, ^67^, ^68^

(7) $$V^{\text{Ewals}}\left(r\right)=\sum_{L_{s}}q_{s}\frac{\mathrm{erfc}\left(\gamma \left|r-L-R_{s}\right|\right)}{\left|r-L-R_{s}\right|}+\frac{4\pi }{v_{c}}\sum_{G\neq 0}\frac{1}{G^{2}}e^{-G^{2}/4\gamma^{2}}\left[\sum_{s}q_{s}e^{iG\left(r-R_{s}\right)}\right],$$

where ***L*** and ***G*** are the real and reciprocal space lattice vectors, *q*_s_ are the charges of each lattice site s of the unit cell at positions *R*_s_, *γ* is the Ewald constant, and *v*_c_ is the volume of the unit cell. A singularity arises at *r* = *R*_*i*_, which is corrected by replacing the ***L*** = 0 and s = *i* case of the first term of Equation (7) with $-\frac{2\gamma q_{i}}{\sqrt{\pi }}$.

Klintenberg, Derenzo, and Weber have developed a method to fit an array of approximately 10,000 point charges to match the Ewald potential in a cluster region of a crystal.^17^, ^18^, ^54^ The charges are distributed in a supercell and fitted with a system of linear equations to match the evaluated Ewald potential at the atomic sites of the central region.

Furthermore, the overall dipole moment of the charges and their total value are constrained to 0. The algorithm is detailed in full in refs 17 and 18. The Ewald program^18^ which implements this algorithm has been modified to allow for the use of noninteger charges and redistributed at https://github.com/Crespo-Otero-group/Ewald with permission from the original authors, fulfilling the requirements of the Computer Physics Communications license.

The array of Ewald‐fitted charges can be used in the electrostatic embedding of $E_{\mathrm{QM}}^{\mathrm{EE}}\left(1\right)$ in Equation (6), thereby supplying the environmental Coulomb interaction of any range order to the excited state region. This extension of the ONIOM Embedded Cluster (OEC) model, is called the ONIOM Ewald Embedded Cluster model (OEEC). Note that the point charge array is not added to the terms *E*_QM′_(**1** ∪ **2**) and $E_{\mathrm{QM}\prime }^{\mathrm{EE}}\left(1\right)$ as this would increase the computational cost and, provided a fixed Region **2**, only adds a constant term to the total energy throughout any sort of optimization.

Setting up hybrid method calculations is typically a technically tedious task, but as many steps as possible are automated in fromage while retaining the full flexibility of the interfacing programs. The line:

fro_prep_run.py

accompanied with a few input files such as the unit cell and a configuration file, will prepare the template files for the actual calculations to follow. This may include cluster geometry generation, Ewald fitting procedures and self‐consistent population analyses.

Then, if the user is satisfied with their newly generated embedded cluster, the line:

fro_run.py

can perform geometry optimization or single point calculations.

#### 3.3.3 Self‐consistent embedding

A further problem to address regarding electrostatic embedding in ONIOM is that of the mutual polarization between regions. Indeed if the embedding charge values are fixed, they are unable to react as the real charge density would to changes in the electronic structure of Region **1**. Most notably, real excitations are liable to provoking an equilibration process with the environment. In order to reflect such processes, fromage combines the self‐consistent Ewald embedding method by Wilbraham et al.^55^ with the ONIOM QM:QM′ formalism. The method is as follows:

1. Region **1** is embedded in an Ewald point charge array.
2. An excited state population analysis is performed on Region **1**.
3. The resulting fractional charges are redistributed in the unit cell.
4. Go to Step 1 using the charges from Step 3 until the population analysis of Step 2 converges.

The final point charge array is used in the embedding of $E_{\mathrm{QM}}^{\mathrm{EE}}\left(1\right)$ in Equation 6. This is an extreme model where the partial charges of the whole crystal correspond to simultaneous and equilibrated excited states. The procedure can also be performed in the ground state, thus providing a self‐consistent preexcitation charge background. This method is called the self‐consistent OEEC (SC‐OEEC). All embedded cluster models are summarized in Table 2.

**Table 2:** Available embedding models

Acronym	Full name	Description
OEC	ONIOM Embedded Cluster	QM:QM' ONIOM cluster model with the QM region embedded in charges from the QM' region
OEEC	ONIOM Ewald Embedded Cluster	OEC with the QM region embedded in Ewald charges
SC‐OEEC‐S_1_	Self‐Consistent ONIOM Ewald Embedded Cluster S_1_	OEC with the QM region embedded in excited state self‐consistent Ewald charges
SC‐OEEC‐S_0_	Self‐Consistent ONIOM Ewald Embedded Cluster S_1_	OEC with the QM region embedded in ground state self‐consistent Ewald charges

#### 3.3.4 Charge value distribution

OEC, OEEC, and SC‐OEEC calculations all require the distribution of point charges from single molecule population analyses to aggregate geometries or unit cell structures. This is a routine operation for preparing force field calculations, where sets of atomic types are associated to a potential or another with predetermined charge values.^56^, ^57^ The atomic type is usually dependent on its neighboring bonded atoms and the bond types. For QM:QM′ calculations, it would be more attractive to use a broader definition of the atomic type which distinguishes between same function atoms at nonequivalent parts of the molecule.

**Figure 8:**
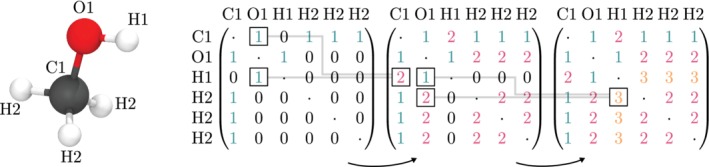
Generation of the bond order matrix for methanol [Color figure can be viewed at http://wileyonlinelibrary.com]

To this end, fromage implements a connectivity detection tool which reads population analysis information from a single molecule or unit cell calculation and redistributes it onto any other finite or periodic ensemble of same molecules. The procedure builds a bond order matrix **B** where *B*_*ij*_ is the shortest path connecting atoms *i* to *j* in number of bonds. The construction of the matrix is represented in Figure 8 and is performed as follows:

1. Detect the first connections by computing all of the interatomic distances complemented by atomic radii.
2. Check every zero element and detect atom pairs (*a*, *b*) which have a connected atom *c* in common, that is, *B*_*ac*_ = 6 ∧ *B*_*bc*_ = 0.
3. Assign *B*_*ab*_ = *B*_*ac*_ + *B*_*bc*_.
4. Repeat from Step 2 until convergence of the matrix.

An atom's identity can be completely defined by additionally using the element of the atom corresponding to each row of **B**. A sufficient fingerprint for a given atom is the amount of atoms of a given element which are located a certain amount of bonds away, accompanied by the atom's own element. For example, a methyl hydrogen of methanol is sufficiently defined by stating that it is a hydrogen atom with one carbon atom one bond away, one oxygen atom two bonds away, two hydrogen atoms two bonds away, and one hydrogen atom three bonds away.

Once all of the atomic identities are sampled from a reference molecule or unit cell, the partial charge values from this reference are distributed onto the desired target. When several reference atoms have the same identity (for instance three hydrogens belonging to the same methyl group), the average charge is retained. In practical terms, given for instance a Mulliken population analysis of methanol calculated in Gaussian with output file pop.log, and a Cartesian coordinate file of a cluster of methanol molecules clust.xyz, the line:

fro_assign_charges.pypop.logclust.xyz

will produce a file out char which contains the coordinates of clust.xyz with an added column stating the partial charge of each atom.

#### 3.3.5 Geometry optimization

Equation 6 provides the energy of the central molecule embedded in its environment. It can readily be extended to its energy gradients, thus allowing for the optimization of geometries. fromage uses the BFGS algorithm as implemented in scipy^16^ to locate ground and excited state minima.

Other critical regions of the PES also play a role in determining the emissive behaviour of the material. When accessible, conical intersections can act as ultrafast funnels producing a nonradiative decay mechanism. It is therefore desirable to locate minimal energy conical intersections (MECIs) and assess their energetics and accessibility. In performing this search, the penalty function method^58^ is employed to avoid the need for nonadiabatic coupling vectors which are not necessarily available depending on the quantum mechanical method employed. The quantity to be minimized is then:

(8) $$F={\bar{E}}_{1-0}+\sigma \frac{\Delta E^{2}}{\Delta E+\alpha },$$

where ${\bar{E}}_{1-0}$ is the average of the *S*_1_ and *S*_0_ energies *ΔE* the energy gap, *σ* is a Lagrangian multiplier, and *α*is a parameter much smaller than *ΔE*. While multireference methods should be used in order to obtain a formal topology of the conical intersection, MECIs obtained with single‐reference methods have proven to also yield informative results at a more modest computational cost.^59^, ^60^, ^61^, ^62^

#### 3.3.6 Example of use

The optimization of excited state geometries in detailed condensed phase environments has numerous applications. For instance, the fluorescence of HC (see Section 3.1.1) can also be switched off by the substitution of a methyl group in *para* position with respect to the hydroxyl group, resulting in another dark compound: (2E)‐3‐[4‐(dimethylamino)phenyl]‐1(2‐hydroxy‐5‐methylphenyl)‐2‐propen‐1‐one (DMAP).^26^, ^27^

Both HC and DMAP can experience excited‐state intramolecular proton transfer, splitting the excited state into two potential decay pathways. It was recently computationally shown that in HC, both the enol and the keto nonradiative decay pathways were rendered energetically inaccessible by a combination of molecular and crystalline factors.^19^, ^63^

**Figure 9:**
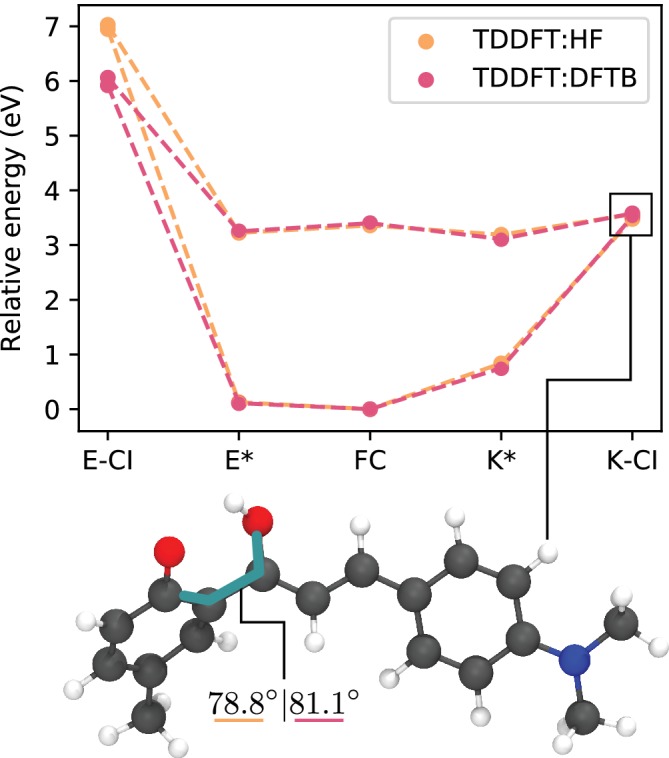
Relative energies of critical geometries of DMAP evaluated with OEEC at critical points of the PES. The Franck–Condon point (FC), Enol (E*), and Keto (K*) excited state minima and conical intersection (CI) geometries were located. DMAP, (2E)‐3‐[4‐(Dimethylamino)phenyl]‐1(2‐hydroxy‐5‐methylphenyl)‐2‐propen‐1‐one; OEEC, ONIOM Ewald Embedded Cluster model; PES, potential energy surface [Color figure can be viewed at http://wileyonlinelibrary.com]

OEEC was employed to investigate the critical points in the PES of DMAP. Geometry optimizations were carried out to locate the ground and excited state minima, as well as its MECI geometries. The crystal structure was optimized in Quantum Espresso using PBE‐D2, a 40‐Ry basis cutoff and a 1 × 2 × 1 *k*‐point mesh. For the ONIOM calculation, the QM level of theory was TD‐ωB97XD/6–311++G(d,p) while for the QM′ level of theory, both HF/STO‐3G and Density Functional Tight‐Binding (DFTB) were employed. The energies and gradients were computed in Gaussian16 for TDDFT and HF and in DFTB+ for DFTB. Figure 9 shows the relative energies of all the optimized critical points of the PES.

The enol nonradiative decay pathway is significantly above the absorption energy, rendering this channel inaccessible. On the other hand, the keto conical intersection is only about 0.2 eV above the absorption energy, arguably making it accessible via thermal fluctuations. The availability of this nonradiative decay channel explains the low fluorescence quantum yield.

The TDDFT:HF and TDDFT:DFTB levels of theory have similar results, both in geometry and energy, with a difference of less than 0.1 eV at each point except form the enol MECI. This outlier can be attributed to the extreme bond stretching occurring between carbons in the back bond in the enol MECI conformation which would render both the Hartree‐Fock formalism and the parametrization of the DFTB calculations inadequate in differing ways. The striking agreement between the two methods is encouraging, given the relatively low computational cost of the semiempirical computation and the prevalence of HF as a ground state theory in QM:QM′ protocols.^50^, ^64^, ^65^, ^66^

## 4 CONCLUSIONS

We have detailed the principal capabilities of the Python library fromage, which aims to facilitate the computational investigation of the excited states of molecular aggregates. They include geometrical and exciton analysis as well as QM:QM′ geometry optimization tools, which have already been successfully employed in three past publications.^12^, ^13^, ^14^ The features were tested on a diverse array of molecules, in order to challenge their robustness. They are implemented with enough flexibility that Python literate researchers can employ fromage scripts as part of a larger workflow with little added effort. By virtue of being an open source, unit tested and documented piece of software, fromage represents an addition to the fast expanding pool of sustainable chemical software libraries. We hope that by enabling researchers to use the framework and manipulate the source code, the field of aggregate photochemistry will continue to mature towards modern reproducible workflows.
